# Modeling tumor evolutionary dynamics

**DOI:** 10.3389/fphys.2012.00480

**Published:** 2013-02-14

**Authors:** Beatriz Stransky, Sandro J. de Souza

**Affiliations:** ^1^Center of Engineering, Modeling and Applied Social Sciences, Federal University of ABCSão Paulo, Brazil; ^2^Ludwig Institute of Cancer ResearchSão Paulo, Brazil; ^3^Institute of Bioinformatics and BiotechnologySão Paulo, Brazil

**Keywords:** modeling, somatic mutations, tumor, tumorigenesis, mutation

## Abstract

Tumorigenesis can be seen as an evolutionary process, in which the transformation of a normal cell into a tumor cell involves a number of limiting genetic and epigenetic events, occurring in a series of discrete stages. However, not all mutations in a cell are directly involved in cancer development and it is likely that most of them (passenger mutations) do not contribute in any way to tumorigenesis. Moreover, the process of tumor evolution is punctuated by selection of advantageous (driver) mutations and clonal expansions. Regarding these driver mutations, it is uncertain how many limiting events are required and/or sufficient to promote a tumorigenic process or what are the values associated with the adaptive advantage of different driver mutations. In spite of the availability of high-quality cancer data, several assumptions about the mechanistic process of cancer initiation and development remain largely untested, both mathematically and statistically. Here we review the development of recent mathematical/computational models and discuss their impact in the field of tumor biology.

## Introduction

Since observations of aberrant mitosis in cancer cells by David von Harsermann, in the late nineteenth century and the proposal that chromosomal abnormalities are fundamental to cancer development by Theodor Boveri in early twentieth century, it is well-established that cancer is a genetic disease of somatic cells. During this period, experimental support of the genetic hypothesis came from the evidence that ionizing radiation and some chemical components, already known to be carcinogenic, could act as mutagenic agents. According to Knudson (2001), the term “somatic mutation” was first applied to cancer by Ernest Tyzzer in 1916, many years before the elucidation of the role of DNA in heredity. But it was only in 1976 when Peter Nowell convincingly stated that cancer is a clonal disease subject to Darwinian evolution, a concept that is now currently established (Nowell, 1976). Two interesting discussions of the initial theories of cancer can be found in the books of Weinberg (2006) and Frank (2007).

Since then, our understanding of the molecular mechanisms underlying tumor initiation and development, involving many specific genes and si pathways, has improved. For example, Hanahan and Weinberg (2000) have suggested that acquisition of six types of molecular alterations, or hallmarks—(1) self-sufficiency of proliferative signals, (2) insensitivity to anti-growth signals, (3) evasion of apoptosis, (4) limitless replicative potential, (5) sustained angiogenesis, and (6) active invasion and metastasis, would progressively drive a population of normal cells to become a cancer (Hanahan and Weinberg, 2000). Crucial to the development of these molecular alterations is genome instability, which generates genetic diversity, which in turn, can further contribute to cancer development (Loeb, 1991). Recently, the same authors added two other features to these hallmarks: the capability to reprogramming energetic metabolism and evading immune surveillance (Hanahan and Weinberg, 2011).

The elucidation of the above mechanisms strengthened the view of cancer as a product of a Darwinian evolutionary process, i.e., a somatic cellular selection, in which (1) the cellular population presents a range of inheritable genetic variations, caused by random mutational processes, and (2) natural selection, where faster dividing cells have an advantage over their normal neighbors. At population level, somatic cells proliferate in an unconstrained manner and compete for nutrients and space. At molecular level, the genome accumulates somatic mutations that allow the cell to reproduce more efficiently and the selection of cells with a higher fitness drives tumorigenesis (Nowell, 1976). Clonal selection is a key concept in tumor biology since it can affect not only replication rate and survival but also invasion capacity and resistance to therapies. Experimental evidences obtained in the last 40 years and the development of (next generation) sequencing technologies—that allowed identification of virtually all genetic alterations in a cancer cell, have stimulated the use of evolutionary-based approaches in the study of cancer. First, clonal expansions and retractions indeed occur within tumors [recently reviewed by Greaves and Maley (2012)]. Second, tumors have been characterized as highly heterogeneous both at the phenotype and genotype levels (Wood et al., 2007; Jones et al., 2008; Parsons et al., 2008). Third, recent studies have confirmed that metastatic lesions are expansions of clones derived from the primary tumor (Jones et al., 2008; Yachida et al., 2010).

Somatic mutations in any cellular genome, either induced by external source or spontaneously occurring during the mitotic replication, may comprise different types of DNA alterations. These include substitutions, insertions or deletions of one base (single nucleotide polymorphism), or segments of DNA; rearrangements; amplification, or deletions that sometimes may comprise the whole genome (polyploidy). In addition, the cell genome may acquire entire sequences from exogenous sources, and epigenetics changes, which alter chromatin structure, can also be subject to the same selection forces as genetic events. However, not all mutations in the cell genome are directly involved in cancer development and it is likely that most of them do not contribute in any way. To relate these capabilities, the terms “driver” and “passenger” mutations were coined (Stratton et al., 2009). A driver mutation is directly implicated as the cause of the tumor process—it confers a growth advantage to tumor cells and is positively selected in the environment of the tissue where the cancer appears. Conversely, a passenger mutation does not confer growth advantage and not contribute to the development of cancer. Today, one of the great challenges is cancer biology is to discriminate between driver, which are potential targets for therapy, and passenger mutations (Beerenwinkel et al., 2007; Wood et al., 2007; Jones et al., 2008).

The availability today of large datasets on different aspects of tumor biology has provided opportunities for the use of mathematical models in oncology, which in turn has provided a more quantitative understanding of the dynamics of tumorigenesis.

## Mathematical modeling

Although mathematical modeling has had a primary role in other areas of science, like physics and engineering, the application of mathematical techniques for generating insights into biological problems has been far less common. Nevertheless, this scenario is beginning to change, with the application of methods from “hard science”—i.e., statistics, computational and mathematical techniques, control engineering, and dynamic systems analysis to address biological data and questions. The mathematical investigation of cancer has not had a very long history—it started in 1950s, aiming to derive the “basic laws” regarding the tumor dynamics, to organize information and to elaborate a comprehensive framework for hypothesis testing (Komarova, 2005; Attolini and Michor, 2009; Byrne, 2010; Vineis et al., 2010). The acknowledgment of these elementary principles can improve our understanding of tumor biology and ultimately can be used to interfere on the mechanisms of tumor initiation, development, treatment, and resistance, in the new area of translational medicine.

Broadly, these models can be organized in three major areas: (1) epidemiology and statistical models, (2) mechanistic growth models, and (3) evolutionary dynamics models (Table 1). The first ones use statistics and incidence curves to create models to explain population data. The carcinogenesis process is then explained by a series of distinct genetic events that cumulates on the tumor development. The second approach uses biological knowledge and a variety of methods to model tumor dynamics, from molecular mechanisms to population level. The carcinogenic process is also understood as a series of distinct events, but those are expanded to epigenetics and genome instability as well. The third group looks at cancer progression within an ecological and evolutionary biology approach, and nowadays is heavily based on cancer genomic data. Obviously, this is just a way to categorize the models, and there is a temporal and conceptual overlap among them. For example, the tumorigenic progress through multiple discrete stages is a common underlying mechanism in all models. However, they are different in the way they explain tumor development. In the first model, the occurrence of a mutational event would be a sufficient condition, while on the third model, this would be just a necessary but not sufficient condition. Therefore, we define a model as a theory established based on observations and premises that incorporates explanatory mechanisms. Generally, a well-constructed theoretical model encompasses interesting biological insights and a reasonable predictive power, and it is based on experimental research and observations. Here we will briefly discuss two distinct approaches, focusing on epidemiological and evolutionary models. This is not intended to be an exhaustive review of the subject, but rather a focused discussion on a limited number of papers in order to illustrate how theoretical representations can assist the improvement of biological knowledge.

**Table 1:** **Theoretical models on carcinogenesis**.

	**Epidemiological**	**Mechanicistic**	**Evolutionary**
Data	Statistics (incidence curves, epidemics)	Experimental (molecular to populational)	Genomic
Biological mechanism—main focus	Multiple distinct genetic events	Genetic and epigentic events, genome instability	Adaptive advantage, “Darwinian selection”
Background	Multi-stage model	Multi-stage model	Evolutionary dynamics
Main reference	Armitage and Doll (1954, 1957)	Several	Beerenwinkel et al., 2007; Bozic et al., 2010

## The “stage models” of cancer—experimental and epidemiological data

Early models that aimed to explain the carcinogenic process were based on experimental and epidemiological data. In the beginning of the twentieth century, experimental studies were usually performed in animal models to identify chemicals that were able to cause cancer. Compounds with strong carcinogen effects like PAHs (polycyclic aromatic hydrocarbons) were painted on the animal skin and tumor growth was analyzed. The researchers observed that the sequential application of two distinctive carcinogens frequently produced a greater ratio of cancer development than the application of an isolated one. In a series of studies, Berenblum and Shubik tested the idea that two distinct tumor phase were stimulated by carcinogen—the “initiation” and “promotion” stages, and in 1949, after experimental verification, they proposed the “Two-stage” model of carcinogenesis (Berenblum and Shubik, 1949). In general, the initiators were the compounds that predispose the tumor development—and for this reason, were considered mutagenic, while promoters were the non-reactive compounds (non-mutagenic)—considered mitogenic, stimulating cell cycle, and tumor growth. Although this theory could not be extensively verified experimentally, the two-stage theory provided the first evidence in favor of the idea of multiple stages of cancer progression.

Later on, another type of evidence, based on epidemiological data of cancer mortality, allowed the development of the multistage theory (MST) of cancer development. Epidemiological studies indicated that (1) cancer incidence often increased rapidly with age, (2) simple patterns could be observed at the population level, although what happened to any particular individual appeared to be highly stochastic. This age-specific uniformity contrasted with the diversity of genetic and environmental mechanisms underlying carcinogenesis, such as the inheritance of genetic variants and exposure of carcinogenic agents. The challenge was to elucidate the essential mechanisms that gave rise to the cancer process by observing the population data, and try to determine to which extension each one of these factors could modify the quantitative properties of age-specific incidence.

Many studies attempted to derive the dynamics of cancer progression from epidemiological data (Fisher and Hollomon, 1951; Nordling, 1953; Stocks, 1953) and indeed they supported the idea that carcinogenesis would require “n” mutational steps. Extending these studies and analyzing incidence curves for a variety of non-endocrine carcinomas, which showed that tumor incidence increased with the sixth power of age, Peter Armitage and Richard Doll, proposed the “Multistage Theory” (MST), in 1954 (Armitage and Doll, 1954, 1957). The theory stated that cancer could arise from normal tissues through a series of multiple stages, assuming that (1) the changes of state are discrete, (2) each stage is stable, and (3) the changes must proceed in a unique order. The theory also explained the linear relationship between carcinogenesis dose and incidence and the long time delay between carcinogen exposure and transformation, observed in experimental studies. Moreover, the model indicates that cancer incidence would increase approximately with a power of age, *t*^*n*−1^, where *t* represents age and *n*, the number of rate-limiting carcinogenic events required for transformation. Fitting the formula *I*(*t*) = k*t*^*n*^—where *I* is the age-specific incidence and *k* is a constant of proportionality, to incidence curves for most of the common adult cancers, Armitage and Doll suggested that *n* should account to 6–7 distinct events. These successive events are biological changes (not necessarily mutations) that give rise to a number of cells with a significant growth advantage, which will develop in an apparent tumor—excluding other processes that could lead to invasion and metastasis. In theoretical models, the term mutation or “event” is usually applied to any genetic modification—such as point mutation, insertion, deletion, chromosome rearrangement, mitotic recombination, and loss or gain of whole chromosome or parts of it—but it can be used to denote a non-mutagenic events as well.

The importance of a model can be measured by the confirmation of its predictions and several incidence curves were observed to match the dynamic predict by MST—about seven steps and constant rates of transition, providing an excellent description of cancers of the colon, stomach, and pancreas. Nevertheless, other tumors, including breast and prostate cancer, failed to fit this model, indicating a different type of tumor progression dynamics—with fewer events and higher rates of limiting events. Two studies, from Ashley (1969) and Knudson (1971), additionally provided an empirical evidence for the MST, although they modified the original conception of multistage model, to incorporate new observed conditions. The rational was: “if somatic mutation is the normal cause of progression, then individuals who inherit a mutation would have one less step to pass before cancer arises” (Frank, 2007). In the log-log plot representation, one less step shifts the incidence curve to earlier ages and reduces the slope. The analysis of the cancer incidence in normal individuals and individuals carrying a mutation that could lead to colorectal cancer was performed by Ashley (1969), and the results confirmed the model hypothesis—predisposed individuals presented cancer in earlier ages. Few years later, Knudson (1971) observed the same shift on the incidence of retinoblastoma, when he analyzed individuals with inherited and non-inherited mutations—and proposed the concept of the tumor suppressor gene (TSG). Cancer would emerge when both alleles of TSG are inactivated. In the first group of children, one allele is already inactivated in their germ line, while the second allele is inactivated by somatic mutation. This is known as Knudson's two-hits hypothesis: it takes two hits to inactivate a TSG. Beyond those models, the MST has been extensively refined, in order to fit data from different bases and assumptions (Armstrong and Doll, 1975; Day and Brown, 1980; Knudson, 2001; Luebeck and Moolgavkar, 2002).

Although Armitage and Doll (1957) have not identified the precise mechanisms underlying carcinogenesis, its major contribution was the development of a conceptual basis that allowed one to relate incidence data, at a population level, to a basic carcinogenic process at the individual level, i.e., the limiting multiple events. It is important to note that the model was proposed in a time when a minimum knowledge about the molecular and cellular mechanisms of cancer was available. This illustrates the importance of good and reasonable assumptions, i.e., the theoretical framework, in elaborating a model. However, without specific knowledge of these factors, we can just estimate the number of crucial events. Actually this is the major drawback of the MST, i.e., its incapacity to link stage identification with the functional changes underlying tumor progression.

This scenario is changing fast, with the development of high throughput technologies, such as genomics and proteomics, which are beginning to provide the complete identification and function of the genetic and molecular machineries in cancer. This knowledge will allow a more precise definition of several parameters that have influenced tumor dynamic—the mitotic and the mutation rates, the type and number of mutational events and the selective forces that occur at each evolutionary stage in a particular type of cancer, leading to refined models of tumorigenesis. Though this represent a good opportunity to develop a comprehensive model of tumor dynamics, it will still be necessary to organize this information, and perhaps the most important goal of high throughput analysis is to determine which changes matter and which are less important.

## Population genetics models—dynamics of genetic variation within population

One of the first mathematical models that explicitly used genome data to simulate the somatic evolution of colorectal tumor was proposed by Beerenwinkel et al. (2007). The model was based on genomic studies of colorectal cancers patients that showed that mutational patterns among those patients were quite diverse—estimating as many as ~20 driver mutations per tumor, and those different landscapes could lead to the same tumor phenotype (Sjöblom et al., 2006). Hence, the model proposed to investigate the importance of selection (the clonal expansion in the model), as the driving force in tumor progression, from a benign tumor (~1 mg or 10^6^ cells) to a full-grown cancer (~1 gr or 10^9^ cells), over a period of 5–20 years. The tumorigenic progression is described as a Wright-Fischer process, where cells evolve in non-overlapping generations and each new cell, generated with the probability proportional to the parental fitness, can acquire a new mutation with “u” probability at each non-mutated gene location. Employing basic parameters—population size (N), number of susceptible genes (potential drivers, k), mutation rate (u) and the fitness advantage (s, always positive), they derive an analytical formula for the expected waiting time for the evolution from benign to malignant stages. As described by Equation (1) (reproduced from Beerenwinkel et al., 2007),

(1) $$t_{k}=k\frac{{\left(\log\frac{s}{ud}\right)}^{2}}{s\log\left(N_{\text{init}}N_{\text{fin}}\right)},$$

the fitness parameter has a major impact on the average waiting time for the tumor manifestation, with the average time reducing approximately as 1/s. In contrast, other parameters like mutation rate and population size have a minor impact—they contribute only logarithmically. Fitting the model to colon carcinomas data, they estimated the number of genes involved as 20 and best estimated for fitness parameter as 0.1, in a very good agreement with the Slobjon study. Although it is widely accepted that the impact of a specific mutation on phenotype will depend on the genetic background, the model assumed that each subsequent mutation has the same incremental effect on the fitness of the cell. It is important to notice that this model does not exclude the presence of highly frequent mutations that confers a large growth advantage to tumor cells, such as APC, K-ras, and p53. Though, it stresses that the pattern of multiple mutations with a small and distinct fitness advantage occurring in dozens of genes will determine and characterize the heterogeneity pattern observed in any kind of tumor. According to this model, tumorigenesis is a dynamical process, where multiple stochastic sequential mutations contribute for the cancer outcome.

Extending these observations, Jones et al. (2008) investigated the common mutational patterns during the phases of tumor initiation, invasion and metastasis, to evaluate the time extending through each phase. Using an approach they called “comparative lesion sequencing” they examined quantitatively the mutations described in colon cancers genomic studies (Wood et al., 2007) and in other neoplastic lesions of the same patients. Hence, they performed a quantitative analysis of data patterns, to estimate the time intervals required for the appearance of the cells that originate the clonal expansions. The basis of the model dynamics can be described as follows: in each carcinogenic stage (microadenoma, small and large adenoma, early and advanced carcinoma, metastasis), there is a founder cell (Fcell) that gives rise to the various tumor populations. During tumor evolution, cells acquire other mutations and might become founder cells of succeeding carcinogenic states. For example, the time interval between the birthdate of a founder cell for a metastasis (Fcell met) and the founder cell of an advanced carcinoma (Fcell aca) can be approximated as Equation (2) (reproduced from Jones et al., 2008).

(2) $$\Delta T_{\text{ACa},\text{Met}}=F_{\text{ACa},\text{Met}}\cdot T_{\text{Met}},$$

An important model premise is the necessity of a constant rate and time for cell divisions over tumor evolution—a requirement that seems to be quite reliable, given the good agreement between the estimated values and the clinical and radiological observations. One of the main predictions is that it takes much longer for a large adenoma to evolve onto an advanced carcinoma than for such carcinoma to metastasize, a result confirmed by simulations where patients data could be applied (patients whom a minimum of 25 mutations could be evaluated). The results also indicated that virtually all of the mutations needed for metastasis are previously settled in all of the cells of the original carcinoma. According to Jones the data analyzed were compatible with two distinct models, “in model A, none of the carcinoma cells can give rise to a metastasis, but they are close to being able to do so; one or a few more genetic alterations are required. In model B, all of the carcinoma cells can give rise to metastasis; no more genetic alterations are required.” Therefore, in a carcinogenic, as well as in a phylogenetic process, mutations act like a clock, providing information about the relatedness of organisms or cells during evolution.

This point was investigated by Yachida et al. (2010), which used genomic data of seven pancreatic cancer patients presenting metastases, to evaluate the clonal relationships among primary and metastatic cancers. The results showed that the pattern observed in the cells that originated metastasis were clearly represented in the cells within the primary carcinoma i.e., itself, a combination of many distinct subclones, each one with its own heterogenic pattern. Additionally, using the mathematical model, they could calculate the elapsed time between the different stages of the tumorigenic process. The results showed an estimation very similar to the one reported by Jones et al. (2008), with an average of 11.7 years from the initiation of tumorigenesis until the birth of the cell giving rise to the parental clone, an average of 6.8 years from then until the birth of the cell giving rise to the index lesion, and an average of 2.7 years from then until the patients' death. Taking these correlations as a conservative assumption, the knowledge of the dynamics of the tumor progression, in quantitative terms, offers a chance to interfere in the tumor evolution and develop a more customized treatment.

**Figure 1:**
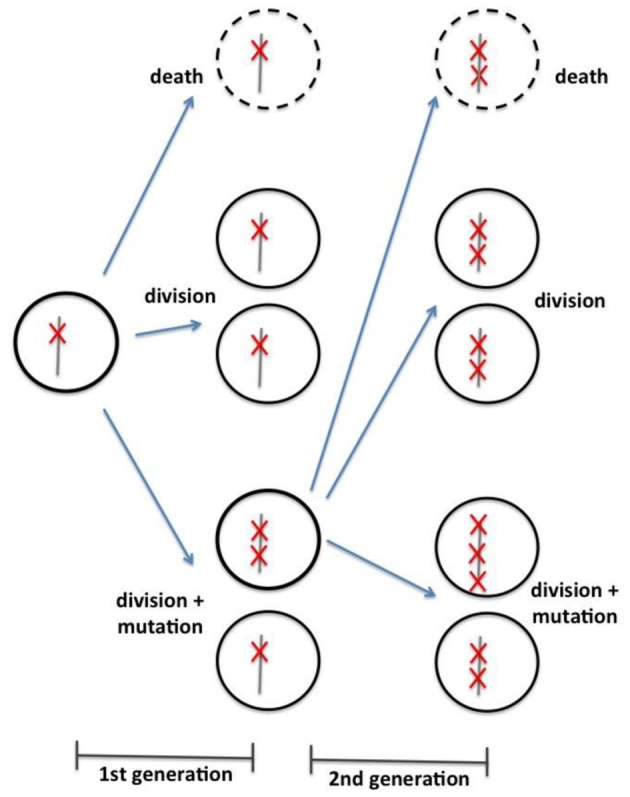
**Schematic representation of branching process model of cell division, as proposed by Bozic et al. (2010).** According to a specific probabilistic distribution, each cell can either (1) die, (2) divide without acquisition of a new mutation, or (3) divide with acquisition of a new driver mutation.

In the same line of investigation as Beerenwinkel et al. (2007) and Bozic et al. (2010) developed a mathematical model to conciliate cancer genomics data with epidemiologic and clinic observations. The model is based on a discrete time Galton-Watson branching process—a stochastic process often used in population genetics. The model emphasis is in tumor progression, not initiation. At each iteration, given a certain probability, a cell can either (1) stagnate (i.e., differentiate, die, or senesce); (2) divide and the daughter cells maintain the same number of driver mutation; or (3) divide and daughter cells receive an additional driver mutation (Figure 1). The simulation begins with a cell presenting a unique driver mutation and it progresses with an increasing rate of clonal expansion, as the cells acquire new driver mutations. The underlying assumption is that a driver mutation reduces the chance for cell stagnation and enhances their “fitness.” Again, the model dynamics depend on only three parameters: the average driver mutation rate (u), the average selective advantage associated with driver mutation (s), and the average cell division time (T). Using an estimated average mutation rate per cell division of 3.4 × 10^−5^, one can calculate the average time between the appearances of successful cell lineages, and therefore, the acquisition of subsequent driver mutations, as shown in Equation (3) (reproduced from Bozic et al., 2010). Moreover, one can also calculate the number of passenger mutations in a particular tumor, taking the selective advantage that a single driver mutation awards to the tumorigenic cell.

(3) $$t_{k}=\frac{T}{ks}\log\frac{2ks}{u},$$

The model was verified both qualitatively and quantitatively by comparing clinical mutation data from glioblastoma multiforme and pancreatic adenocarcinoma, the most common type of tumor in brain and pancreas, respectively, with the data obtained by simulation. Fitting the model parameters to the numbers of driver mutations from clinical data, the average selective advantage of a driver mutation was estimated as 0.4%, for both tumors. This value was confirmed by a best-fit parameter analysis for glioblastoma and pancreatic cancers, using the same mutation rate. Besides, they observed that changing the mutation rate (u) in two orders of magnitude—from 10^−4^ to 10^−6^, resulted in an average selective advantage (v) of 0.65 and 0.32%, respectively. This remarkable consistency of the v parameter seems to represent at least the magnitude of the advantage that the driver mutations confer to a tumor cell, which seems to be very small (0.4%). This has a direct implication on the pace development that is observed within any type of tumor. Since more than 99% of driver mutations become extinct, the probability of a clonal expansion from a single driver mutation is almost null.

Despite the oversimplifications and limiting assumptions, concerning the fixed life spam of tumor cells, the exponential growth patterns of the tumor and specially, the constant fitness value of driver mutations, the model captures several essentials characteristics of tumor growth in a more quantitative scenario. Besides, it puts into perspective the complexity of cancer biology (as any other biological process) with all factors concourse for the tumorigenic process—the cell fitness, i.e., the cell division rate and the grow advantage conferred by mutation, and also the environment selective pressure. As any type of (theoretical) model, there will be always the possibility of refinements as long as the information about the particular system is improved. When the model hypothesis and previsions are in disagreement with the observed data, the model structure should be reevaluated in order to better represent the biological knowledge.

## Prospects

Sequencing technologies will continue to progress and this will affect the way we diagnose and treat tumors in a dramatic fashion. Data generated from thousands, or even millions, of patients will feed the development of more refined mathematical models of cancer. For example, it is reasonable to envisage that soon we will have the genomes of several tumor cells within the same tumor due to the development of whole-genome sequencing strategies for single cells. With that, we will have more precise information on the level of genetic heterogeneity within a tumor. Furthermore, that will allow the characterization, in quantitative terms, of all the evolutionary forces acting on tumor cells in a temporal fashion. For example, it is expected that the frequency of a given passenger mutation will fluctuate within the tumor in a way similar to a neutral mutation in a population, i.e., driven by random drift. That type of information will allow the development of more precise mathematical models.

Another important issue is the integration of more precise clinical data. With cancer genomes being sequenced, associations between genetic and clinical patterns will become more sounded. Ultimately, the goal of any mathematical modeling of the tumorigenic process is to predict what genetic alterations are predictive of any important clinical feature. Associations between these genetic alterations and clinical phenotypes are therefore very important in the mathematical modeling of tumors.

### Conflict of interest statement

The authors declare that the research was conducted in the absence of any commercial or financial relationships that could be construed as a potential conflict of interest.
